# Detection of citrus diseases in complex backgrounds based on image–text multimodal fusion and knowledge assistance

**DOI:** 10.3389/fpls.2023.1280365

**Published:** 2023-11-27

**Authors:** Xia Qiu, Hongwen Chen, Ping Huang, Dan Zhong, Tao Guo, Changbin Pu, Zongnan Li, Yongling Liu, Jin Chen, Si Wang

**Affiliations:** ^1^ Institute of Remote Sensing and Digital Agriculture, Sichuan Academy of Agricultural Sciences, Chengdu, China; ^2^ Science and Technology Center of Intelligent Agriculture, Sichuan Academy of Agricultural Sciences, Chengdu, China; ^3^ State Key Laboratory of Remote Sensing Science, Institute of Remote Sensing Science and Engineering, Faculty of Geographical Science, Beijing Normal University, Beijing, China

**Keywords:** citrus disease, deep learning, multimodal fusion, background diversity, knowledge assistance

## Abstract

Diseases pose a significant threat to the citrus industry, and the accurate detection of these diseases represent key factors for their early diagnosis and precise control. Existing diagnostic methods primarily rely on image models trained on vast datasets and limited their applicability due to singular backgrounds. To devise a more accurate, robust, and versatile model for citrus disease classification, this study focused on data diversity, knowledge assistance, and modal fusion. Leaves from healthy plants and plants infected with 10 prevalent diseases (citrus greening, citrus canker, anthracnose, scab, greasy spot, melanose, sooty mold, nitrogen deficiency, magnesium deficiency, and iron deficiency) were used as materials. Initially, three datasets with white, natural, and mixed backgrounds were constructed to analyze their effects on the training accuracy, test generalization ability, and classification balance. This diversification of data significantly improved the model’s adaptability to natural settings. Subsequently, by leveraging agricultural domain knowledge, a structured citrus disease features glossary was developed to enhance the efficiency of data preparation and the credibility of identification results. To address the underutilization of multimodal data in existing models, this study explored semantic embedding methods for disease images and structured descriptive texts. Convolutional networks with different depths (VGG16, ResNet50, MobileNetV2, and ShuffleNetV2) were used to extract the visual features of leaves. Concurrently, TextCNN and fastText were used to extract textual features and semantic relationships. By integrating the complementary nature of the image and text information, a joint learning model for citrus disease features was achieved. ShuffleNetV2 + TextCNN, the optimal multimodal model, achieved a classification accuracy of 98.33% on the mixed dataset, which represented improvements of 9.78% and 21.11% over the single-image and single-text models, respectively. This model also exhibited faster convergence, superior classification balance, and enhanced generalization capability, compared with the other methods. The image-text multimodal feature fusion network proposed in this study, which integrates text and image features with domain knowledge, can identify and classify citrus diseases in scenarios with limited samples and multiple background noise. The proposed model provides a more reliable decision-making basis for the precise application of biological and chemical control strategies for citrus production.

## 1 Introduction

Citrus crops are among the most important fruit crops worldwide, and they are widely cultivated in more than 140 countries and regions and have significant economic value (Rao et al., 2021). However, citrus pests and diseases pose serious threats to orchard production in terms of quality and yield (Sun et al., 2019) and represent major factors that hinder the sustainable development of the citrus industry. Thus, the development of efficient and applicable methods for detecting citrus pests and diseases is crucial to ensuring the robust expansion of the citrus industry. Previous studies typically identified citrus diseases through field observations (Leong et al., 2022) or pathogen identification (Patané et al., 2019). However, these methods are influenced by subjective factors and require domain knowledge and specialized equipment; further, they often present low accuracy and efficiency. With the advancement of computer vision technology, machine learning methods have gradually been applied to identifying citrus diseases. Initially, researchers explored traditional machine learning algorithms involving commonly used support vector machine (SVM) and random forest (RF) algorithms (Wang et al., 2021c; Dananjayan et al., 2022). Classification and regression trees (CARTs) and multilayer perceptrons (MLPs) have been used to identify storage diseases in citrus (Gómez-Sanchis et al., 2012). However, these methods are not appropriate for complex image features and multicategory classifications; thus, their effectiveness in practical applications is limited. In recent years, significant progress has been made in the use of deep learning technology for citrus disease identification. Convolutional neural networks (CNNs) are representative deep learning methods that have achieved breakthrough results in image classification tasks. Classic and lightweight networks, such as visual geometry group (VGG) (Xing et al., 2019), residual network (ResNet) (Luaibi et al., 2021), and efficient convolutional neural networks for mobile vision (MobileNet) (Barman et al., 2020), have been successively applied to image feature extraction and classification to improve citrus disease classification accuracy through higher-level feature mining. Despite these advances, existing methods face challenges such as insufficient training samples required for higher detection accuracy and poor transferability to complex production environments and diverse disease types. Therefore, improving detection accuracy based on limited sample sizes has become increasingly important.

Deep learning has emerged as a research focal point for the accurate identification of plant diseases. It overcomes the limitations of traditional machine learning, which relies on manually generated features, by enabling the construction of an end-to-end deep network structure. This facilitates an automated process that is advantageous for extracting high-level features (Goodfellow et al., 2016). Previous research on deep learning based methods have achieved promising results in the early detection of plant diseases (Upadhyay and Kumar, 2021), lesion segmentation (Li et al., 2022), disease type classification (Xing et al., 2019), and disease occurrence prediction (Delnevo et al., 2022). Historically, citrus disease identification and classification methods have primarily used single-source data based on image modalities, including images (Barman et al., 2020; Luaibi et al., 2021; Syed-Ab-Rahman et al., 2022), fluorescence spectra (Neves et al., 2023), and Internet of Things (IoT) data (Delnevo et al., 2022). The performance of such methods is highly dependent on large datasets and manual annotation. Expanding datasets to improve disease identification performance can be expensive. Ferentinos (2018) used 87848 images covering 25 plants and 57 diseases and compared the disease identification accuracy of five typical CNN networks; the results showed that the VGG model achieved the highest accuracy of 99.53%. In addition, Chellapandi et al. (2021) and Saleem et al. (2020) used 54306 images of 14 diseases, Abbas et al. (2021) used 16,012 tomato disease images, and Brahimi et al. (2017) used 16012 tomato disease images for training and obtained more than 99% accuracy on networks such as DenseNet, AlexNet, and Xception. However, with smaller datasets, the training accuracy rarely exceeded 95% (Ramcharan et al., 2017; Sibiya and Sumbwanyambe, 2019). In addition, approximately 50% of the data in current plant disease identification research are obtained from public datasets (Ramanjot et al., 2023). Using PlantVillage as an example, images are primarily acquired in laboratories or under unique background conditions. The uniformity of sample backgrounds hinders model feature learning, and uncertainties caused by sample selection biases hinder the adaptation of automated plant disease detection systems production scenarios (Hernandez and Lopez, 2020). Although the aforementioned studies have achieved satisfactory identification results using specific datasets, challenges, there are challenges such as poor model robustness, long model iteration cycles, and difficulty in generating massive datasets. As a result, models cannot easily adapt to complex environments and backgrounds in real-world scenarios.

Given the development of deep learning technologies and the rapid acquisition of multi-source data coupled with complex and varied real-world scenarios involving multiple data types, multimodal fusion technology has been introduced in the field of plant pest and disease detection. The full exploitation of the complementarity and correlations between modalities to achieve multimodal data fusion has emerged as a promising new direction in disease research (Yang et al., 2021). By integrating images with environmental parameters (Zhang et al., 2022), hyperspectral information (Yang et al., 2021), and text information (Wang et al., 2021b), the close relationship between disease occurrence and the environment can be fully exploited. Moreover, the complementarity between modalities facilitates the identification of highly similar symptoms and disease classifications under limited sample conditions. Text data are relatively easy to obtain and can be processed without sophisticated equipment and techniques; thus, they can serve as an excellent source of auxiliary information. Information obtained from text sources can complement that from image sources, thereby alleviating the problem of insufficient image training samples (Wang et al., 2022). In fine-grained image recognition tasks, image and text information are jointly trained through different training forms and feature representations, which effectively addresses the problem wherein the image modality is similarly represented but other modalities are underutilized (Reed et al., 2016; He and Peng, 2020). However, traditional textual information derived from natural language descriptions of observers may be incomplete or erroneous because of subjectivity and limitations in the knowledge background (Wang et al., 2021a). Moreover, the preparation and preprocessing of natural language methods are challenging and time consuming.

Given the needs and weaknesses of existing methods, this study aimed to develop a plant disease detection method based on a deep learning feature fusion network based on a multimodal image-text classification to improve the accuracy and efficiency of citrus disease detection in complex backgrounds. By selecting excellent networks of a single image and text modal information and constructing fusion models, the cross-complementarity of information was fully exploited, which enhanced the comprehensive use of features and improved detection accuracy and robustness. Moreover, we propose the use of agricultural domain knowledge to construct a structured glossary for citrus disease characteristics. This glossary can assist in the preparation of text modal information, increase the efficiency of data production, and improve the credibility of identification results. To evaluate the performance and strengths of the proposed method, we used various sample settings and performed dataset cross-validations.

## 2 Materials and methods

### 2.1 Image and text models used in this article

**Figure 1:**
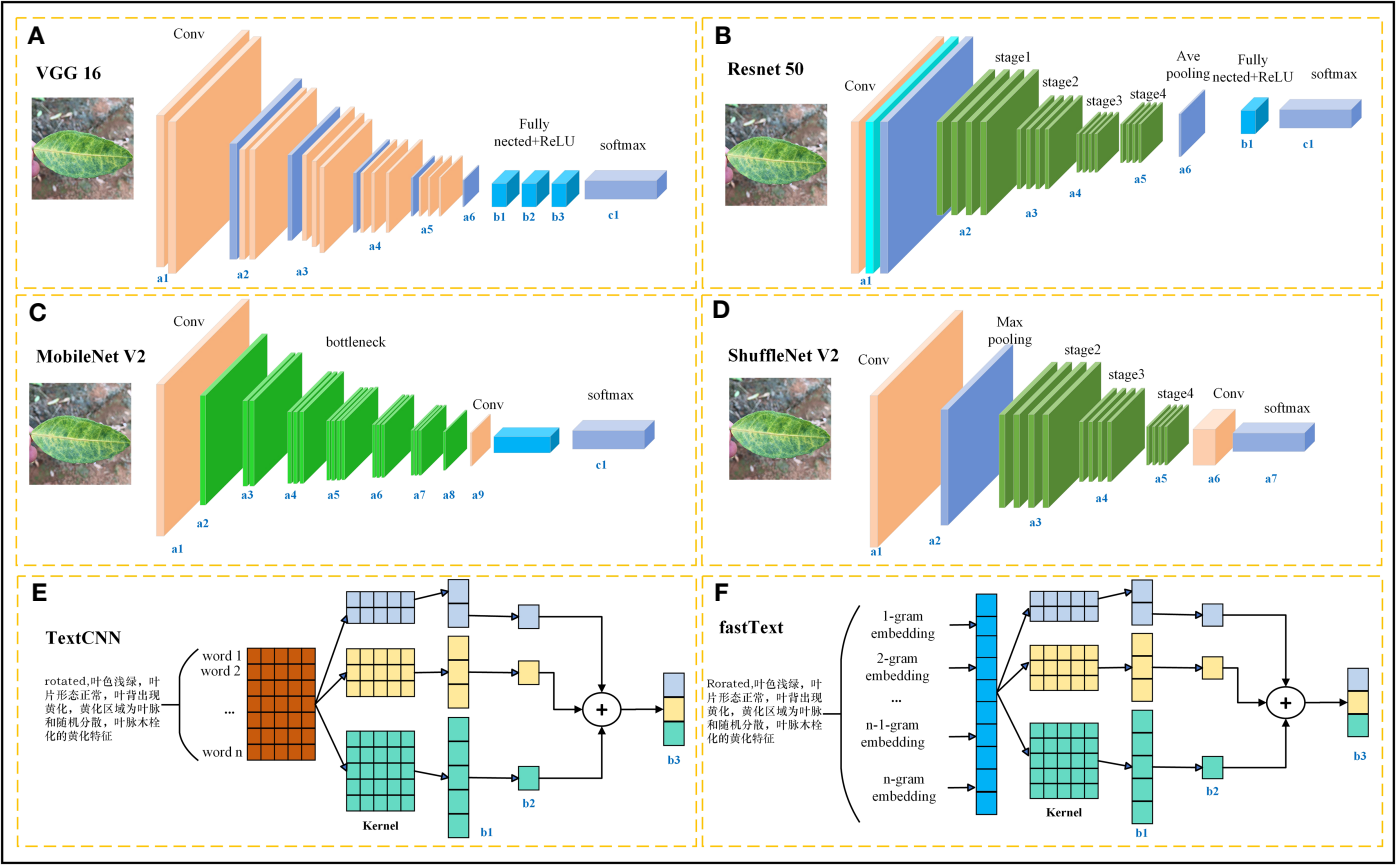
Basic framework of the used image and text models. **(A)** VGG16, **(B)** ResNet50, **(C)** MobileNet V2, **(D)** ShuffleNet V2, **(E)** TextCNN, and **(F)** fastText.

Deep learning architectures are associated with advancements in various domains, including plant disease identification. This section provides a review of the structural features of the models used in this study, as well as their applications and potential advantages in plant disease identification. The basic frameworks of the models are shown in **Figure 1**. VGG16 has 13 convolutional layers and 3 fully connected layers, and it can use small filters and deeper layers to extract more complex features from disease images (Simonyan and Zisserman, 2014). In the context of plant disease identification, VGG16 has been used to extract complex patterns and features from disease images of rice (Jiang et al., 2021), millet (Coulibaly et al., 2019), canola (Abdalla et al., 2019), and tomato (Rangarajan et al., 2018). ResNet50 introduced a residual network structure that effectively avoids overfitting problems by increasing the network depth (He et al., 2016); additionally, it has been shown to provide superior performance in scenarios with complex background noise and diverse disease manifestations (Picon et al., 2019). MobileNet V2 and ShuffleNet V2 stand out as lightweight models. MobileNet V2 uses inverted residuals and linear bottlenecks to enhance efficiency (Sandler et al., 2018), while ShuffleNet V2 employs group convolution and channel shuffling (Ma et al., 2018). Thus, they are ideal choices for resource-constrained environments. These lightweight networks with fewer parameters are efficient and accurate in plant disease classification and suitable for deployment on limited-resource devices (Barman et al., 2020; Lu et al., 2023). In terms of the text modality, this study compared the classification performance of TextCNN and fastText. While TextCNN leverages convolutional layers over word embeddings to discern local semantic features in text (Kim, 2014), fastText captures morphological nuances by representing words through character n-grams (Joulin et al., 2016). Textual descriptions accompanying plant images can provide crucial contextual information. Combining visual and textual modalities can enhance the overall accuracy of disease classification and assist in fine-grained disease categorizations (Wang et al., 2021b). With the advancement of technology, the amalgamation and refinement of these models will further increase the precision and applicability of plant disease identification.

### 2.2 Data preparation

Through field collection, laboratory photography, and web crawling, a total of 2200 citrus image samples were gathered; they comprise healthy leaves, 2 bacterial diseases (citrus greening and citrus canker), 5 fungal diseases (anthracnose, scab, greasy spot, melanose, and sooty mold), and 3 physiological disorders (nitrogen deficiency, magnesium deficiency, iron deficiency). Each sample category contains 100 images with white backgrounds and 100 images with natural backgrounds. Original sample images are shown in **Table 1**.

**Table 1:** Example of the image-text origin database.

Disease category	Origin Image	Origin Text
Healthy leaves (CK)	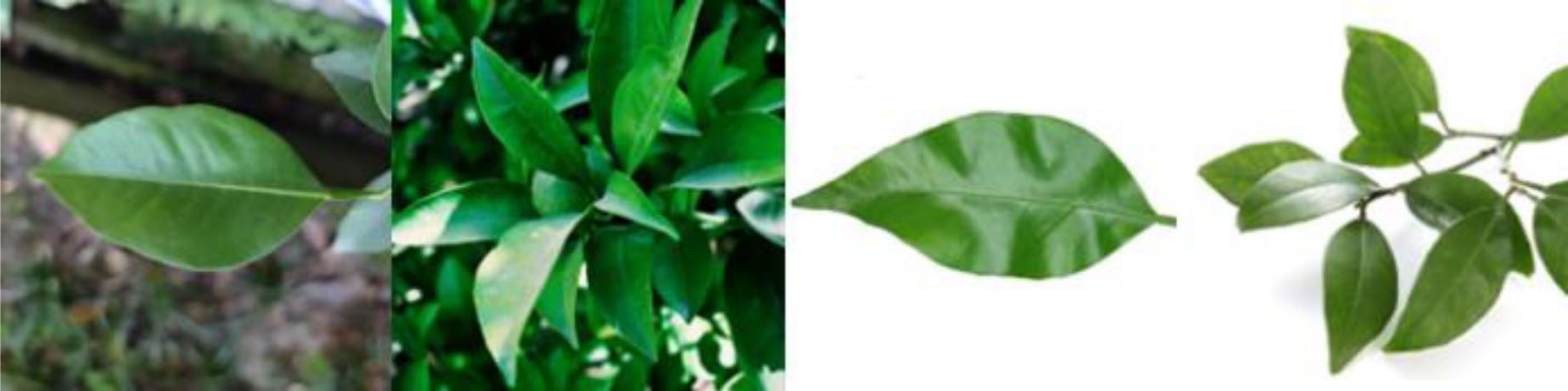	Deep green in color, normal in morphology, asymptomatic on the front side.
Citrus greening (CGR)	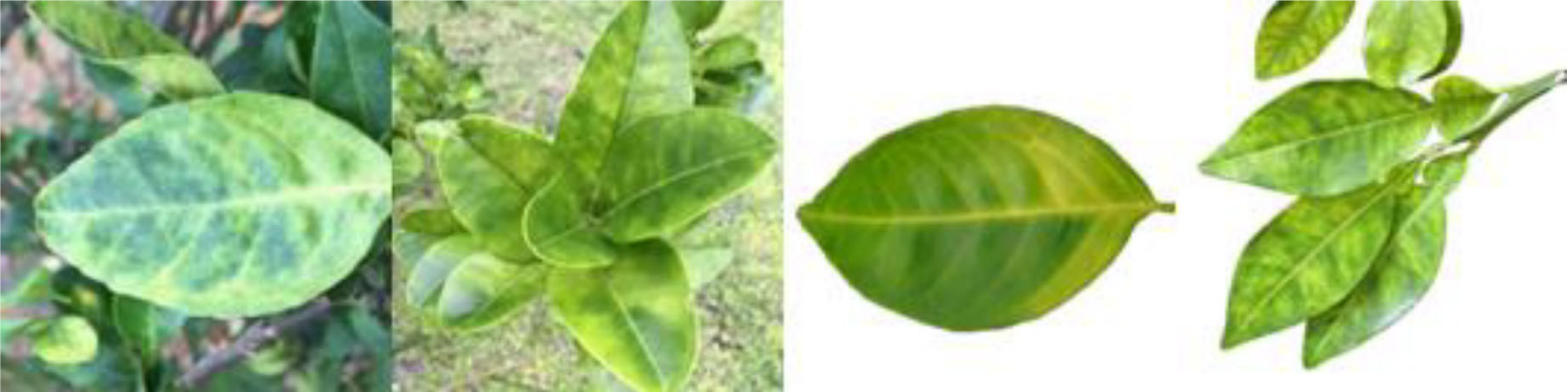	Light green in color, normal in morphology, chlorosis on the back side, chlorosis region is veined and randomly dispersed.
.Citrus canker (CCA)	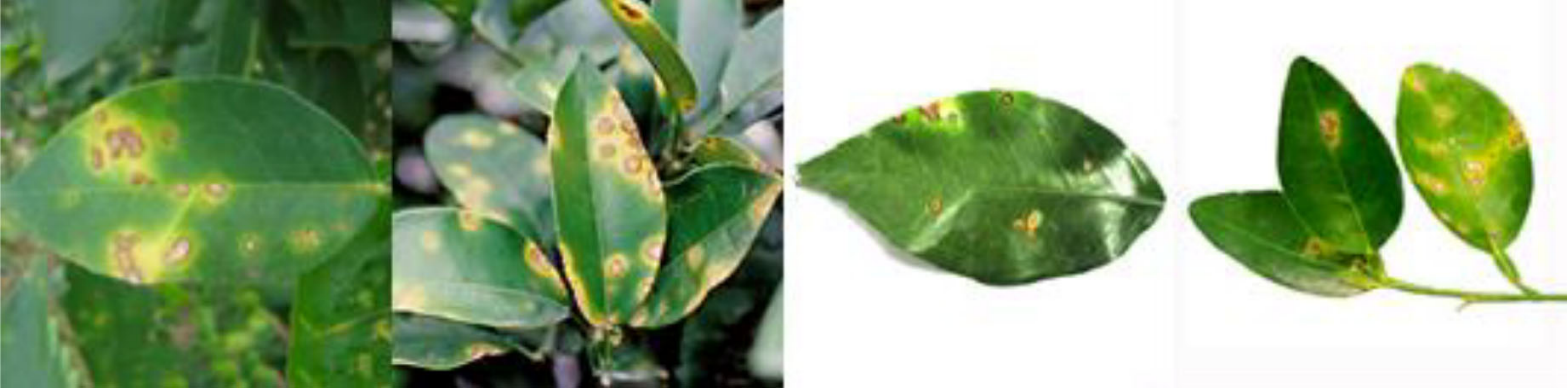	Deep green in color, normal in morphology, lesions on the front side, lesions shape is subcircular, lesions count is 4–20, lesions size is 3–5 mm, lesions are randomly dispersed, lesion color is brown at the center and yellow at the edges, lesions show a volcano-like feature.
Citrus anthracnose (CAN)	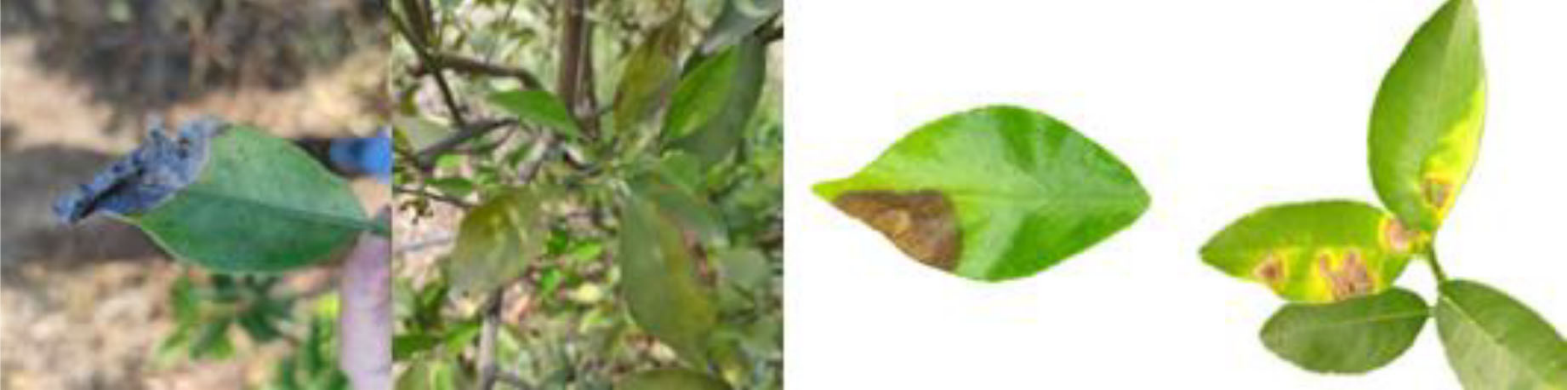	Deep green in color, curled in morphology, lesions on the front side, lesions shape is subcircular, lesions count is less than 3, lesions size is larger than 15 mm, lesions are randomly dispersed, lesions color is light brown, lesions show a withered feature
Citrus scab (CSC)	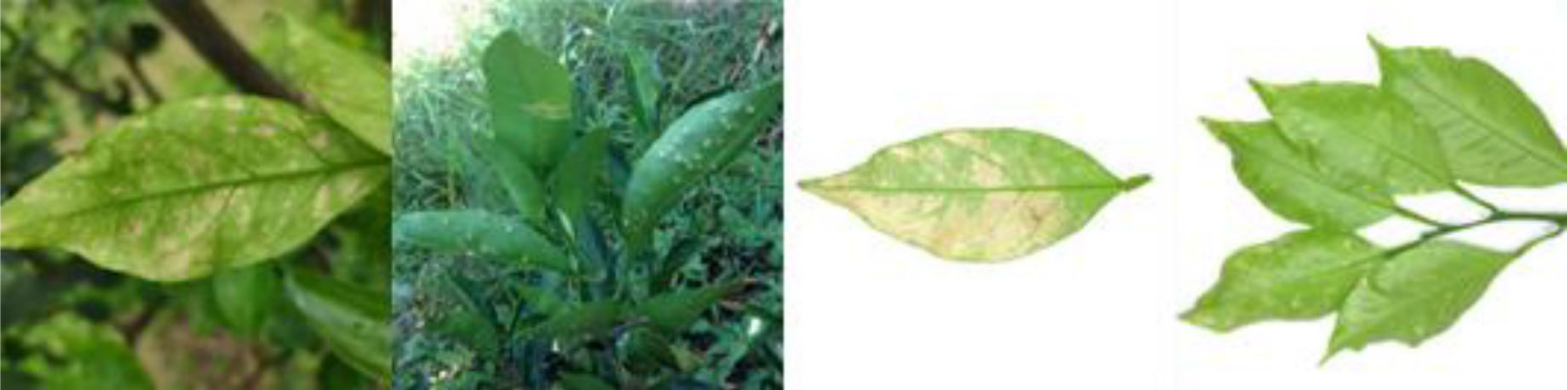	Light green in color, normal in morphology, lesions on the back side, lesions shape is irregular, lesions count is 4-20, lesions size is 3–5 mm, lesions are randomly dispersed, lesions color is gray-white, lesions show a corked feature.
Citrus greasy spot (CGS)	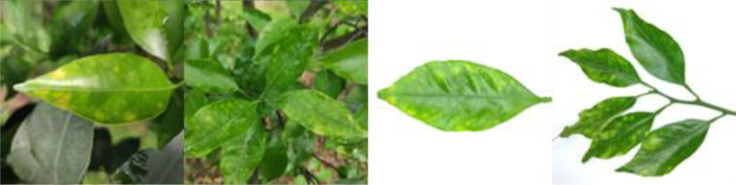	Light green in color, normal in morphology, lesions on the front side, lesions shape is irregular, lesions count is 4–20, lesions size is 3–5 mm, lesions are randomly dispersed, lesions color is yellow, lesions show a flat and greasy feature.
Citrus melanose (CME)	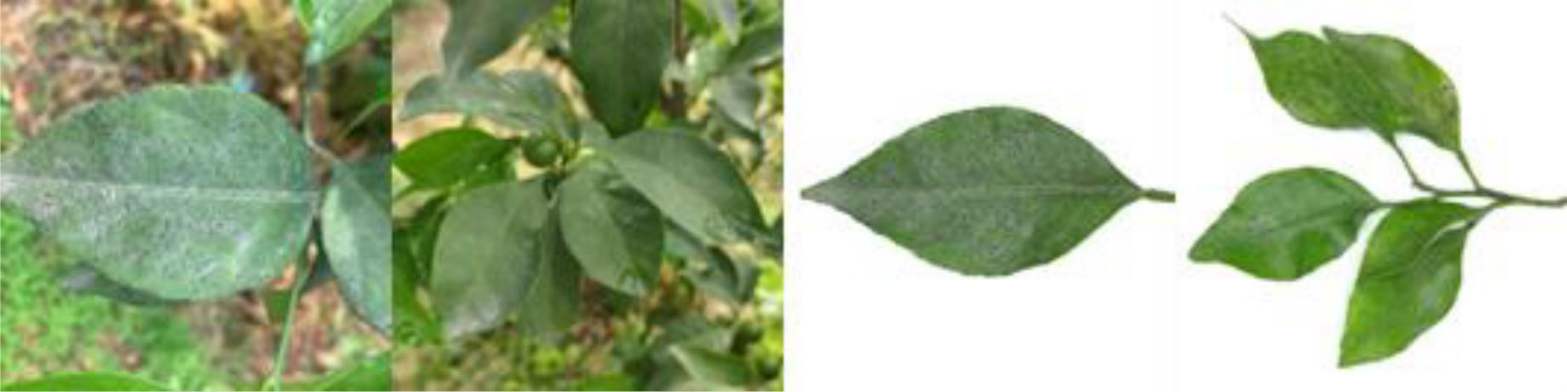	Deep green in color, normal in morphology, lesions on the front side, lesions shape is irregular, lesions count is more than 20, lesions size is 1–3 mm, lesions are randomly dispersed, lesions color is dark, lesions show a convex and greasy feature.
Citrus sooty mold (CSM)	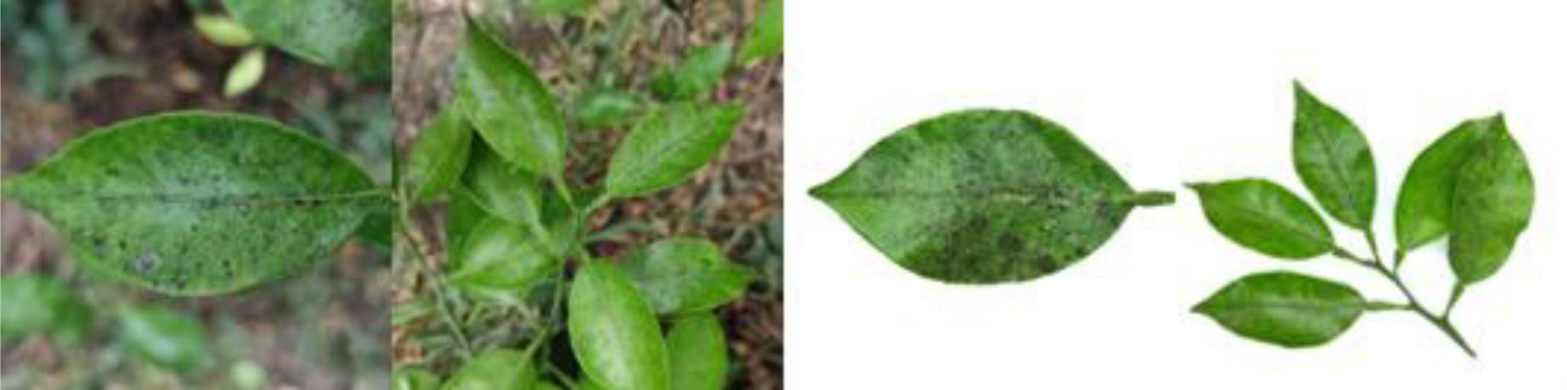	Deep green in color, normal in morphology, black mold spots or layers covering the front side.
Citrus nitrogen deficiency (CND)	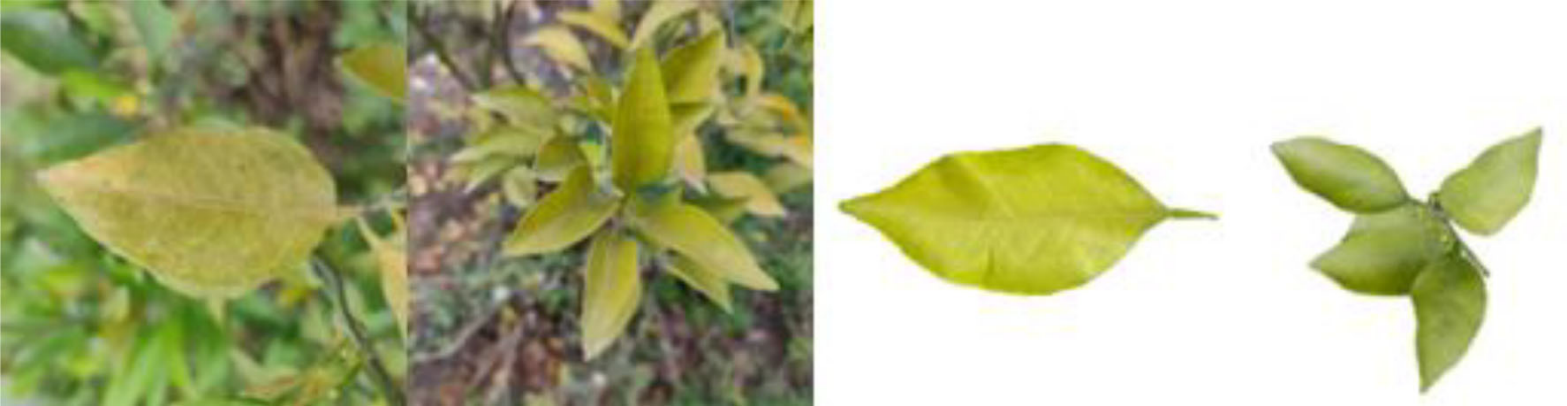	Yellow in color, normal in morphology, chlorosis on the front side, chlorosis region is uniformly dispersed
Citrus magnesium deficiency (CMD)	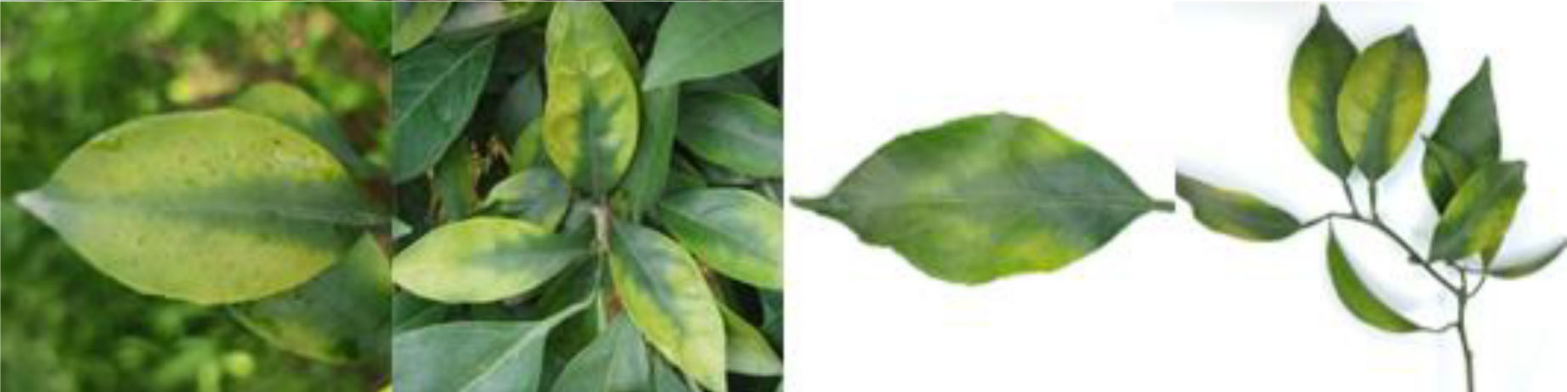	Light green in color, normal in morphology, chlorosis on the front side, chlorosis region near the leaf margin, inverted V-shaped chlorosis
Citrus iron deficiency (CID)	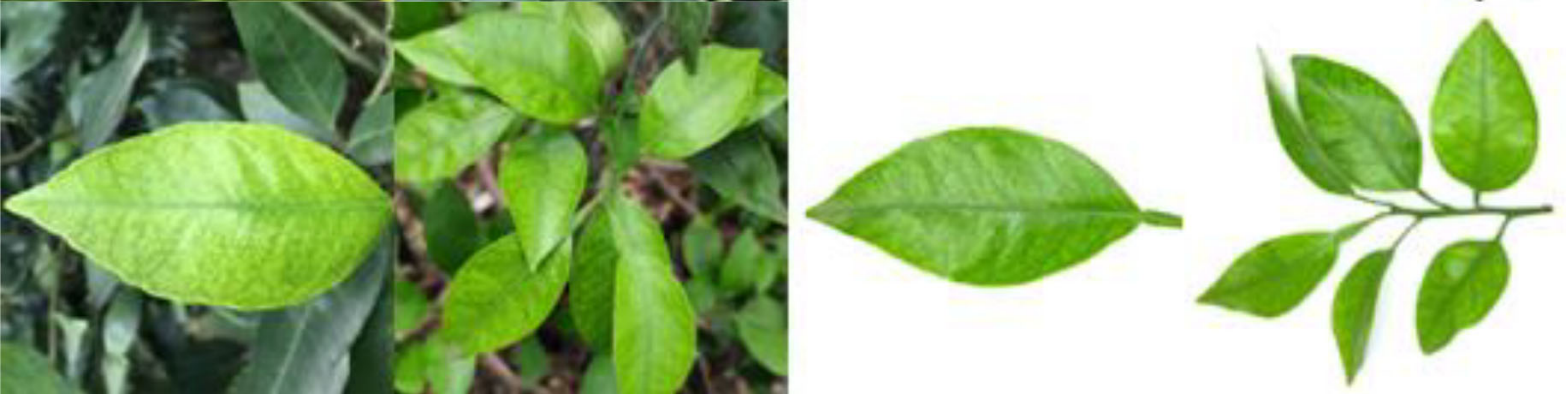	Light green in color, normal in morphology, chlorosis on the front side, interveinal netted chlorosis

In addition to the image data, we used expert knowledge to create a structured citrus disease features glossary (**Table S1**). This glossary covers 12 categories of citrus disease characteristics, including leaf color, leaf morphology, affected areas, covering features, chlorosis region, chlorosis features, lesion shape, lesion count, lesion size, lesion distribution, lesion color, and lesion features. Such data provide a more accurate and comprehensive description of the original images. To ensure data accuracy, the textual data were generated by three Ph.D. scientists specializing in pomology and checked by a psychologist. Sample origin texts are shown in **Table 1**.

### 2.3 Data preprocessing

The original image was uniformly cropped and -adjusted to 224×224 pixels. The image was enhanced by rotation, scaling, flipping, and brightening. Simultaneously, data enhancement methods such as “origin,” “rotated,” “brighter,” “flipped,” and “scaled,” were embedded in the text descriptions to ensure a one-to-one correspondence between the image and text. Symbols from the text were filtered and tokenized using Jieba, and the tokenized results were then mapped to a word index list based on a vocabulary. Words that were not present in the vocabulary were replaced with “<UNK>“. The max_length of the numeric sequence was set to 100, and placeholders were used to supplement any shorter parts. Finally, PyTorch’s tensor conversion was used to transform the numeric sequence into tensors for subsequent calculations. The training, verification, and test sets were divided at a ratio of 7:2:1 for the image–text pairs.

### 2.4 Single-modality comparative experiments

To identify a network architecture that can effectively extract features from citrus disease images, we compared the classification performance of four deep learning networks: VGG16, ResNet50, MobileNet V2, and ShuffleNet V2. These networks use convolutional operations that target key features in images, such as texture, color, and shape, to produce more abstract feature representations. Throughout the training process, all networks used the Adam optimizer and the learning rate was set to 0.001 to ensure training stability and efficiency. This experiment was conducted to comprehensively evaluate the performance of each network in the citrus disease image classification task and provide a basis for selecting image networks for subsequent multimodal construction.

To select a network structure that can effectively extract features from citrus disease text information, we compared the classification performances of two deep learning networks: TextCNN and fastText. Structured text descriptions were converted into word vectors, and text extraction networks were used to extract features such as contextual relationships. The optimizer and learning rate settings were consistent with the image networks described in Section 4.3.1 to ensure training consistency and fairness for comparison. To avoid overfitting, the dropout was set to 0.5 and the length of the input text vector was set to 20. This experiment was conducted to comprehensively assess the performance of each network on the citrus disease text classification task and provide a basis for selecting the text network in the subsequent multimodal construction.

### 2.5 Different-dataset comparative experiments

**Figure 2:**
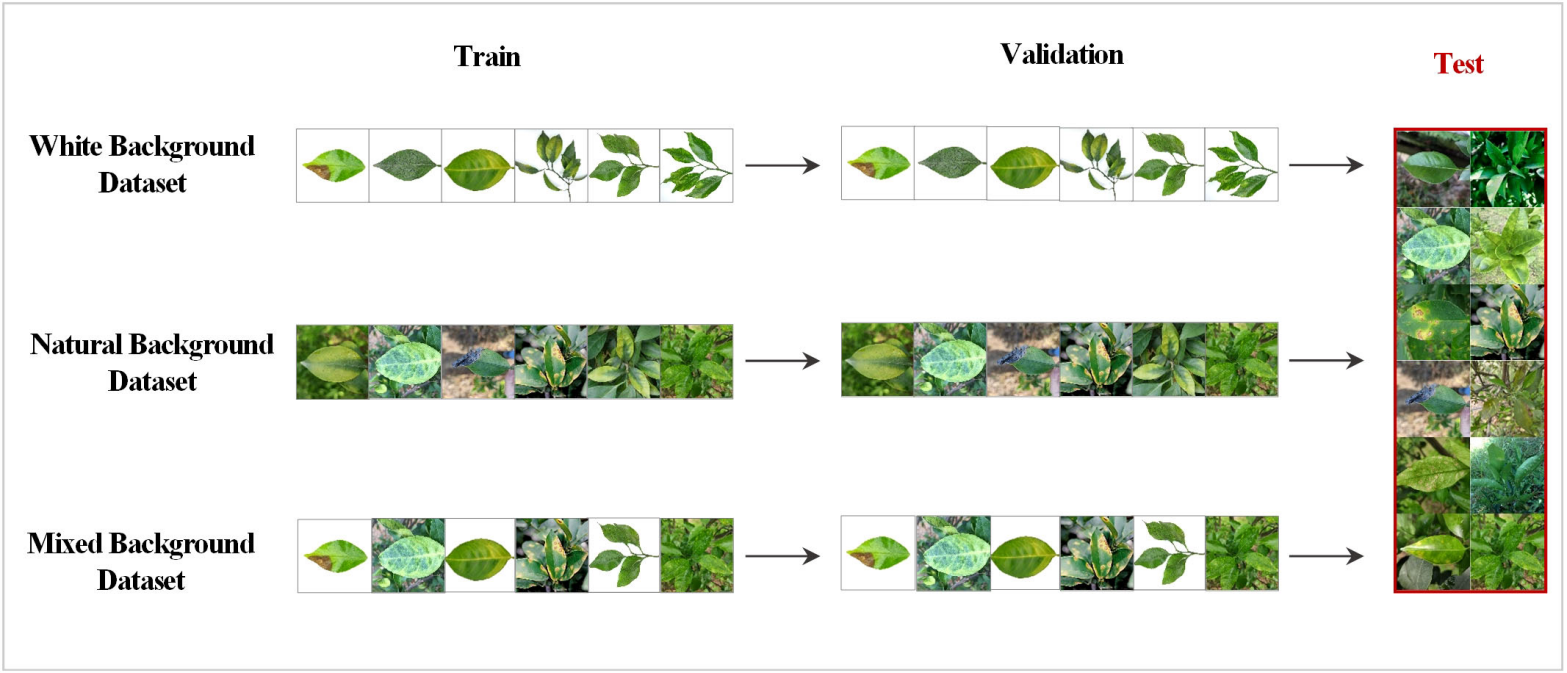
Training and testing strategies for the different datasets; 10% of the natural background dataset was randomly extracted as the shared test set.

To increase the diversity and practical applications of the data, this study created three types of datasets: white, natural, and mixed backgrounds. Specifically, in the white background dataset, each category contains 100 images of single leaves with a white background and multiple leaves with a white background. In the natural background dataset, each category contains 100 images of single leaves with a natural background and multiple leaves with a natural background. The original images in each of the two above datasets were expanded to 5500 images through data augmentation techniques, such as rotation and highlighting. Then 5500 images from these two datasets were randomly obtained in equal proportions to form the mixed background dataset. All three datasets were trained using the selected MobleNet50 and ShuffleNet V2 networks. Testing was always performed using the natural background dataset. The training and testing processes are shown in **Figure 2**.

### 2.6 Construction of the image-text multimodal networks

**Figure 3:**
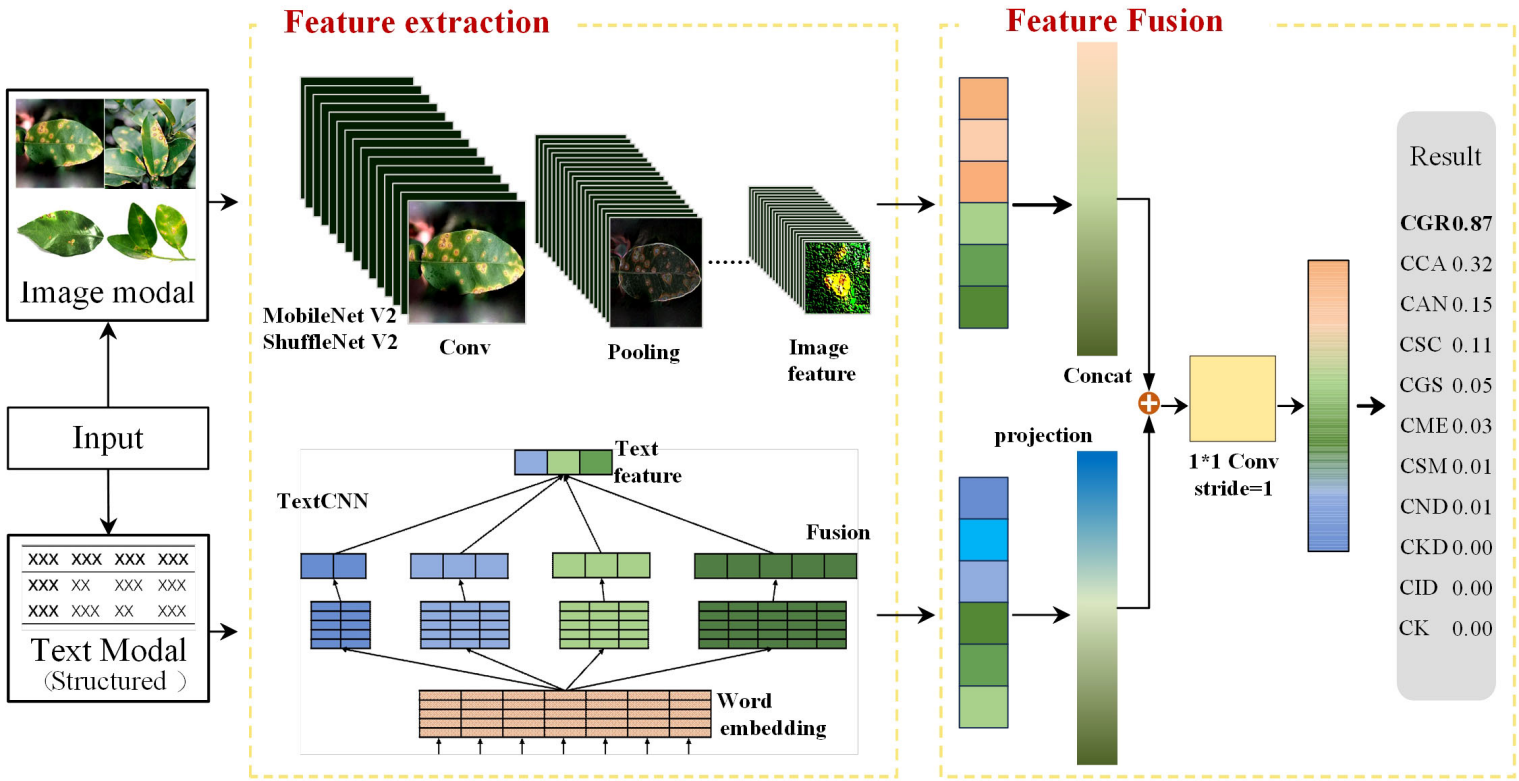
Image-text multimodal network framework.

The image-text feature fusion framework proposed in this study consists of two network branches: MobileNet V2/ShuffleNet V2 and TextCNN. The input data consist of a disease image and structured text describing the disease features. These descriptions were corrected by fruit tree experts to ensure the standardization of the feature description. MobileNet V2 and ShuffleNet V2 extract the image feature vectors from the image-text pairs, while TextCNN extracts the text feature vectors from the data pairs. The two types of feature vectors are concatenated to obtain the feature vector for the image-text pair. The model framework is shown in **Figure 3**.

#### 2.6.1 Image branching in the multimodal framework

The image branch selected two lightweight networks (MobileNet V2 and ShuffleNet V2) that performed well in single-image-modality comparative experiments. MobileNet V2 decomposes traditional convolution operations into depth-wise and point-wise convolution steps, and it also adopts residual connections and dilated convolutions to enhance the expressive capability of models. When training the image modality with MobileNet V2, the input image passes through 2 convolutional layers and 17 bottlenecks to finally obtain the image feature vector. MobileNet V2 introduces an inverted residual module that differs from the traditional bottleneck. It first expands the feature vector dimensionally through an expansion layer and then reduces dimensionally through a pointwise convolution layer, with the expansion factor set to six times. For instance, in the second bottleneck with an input of 112×112×16, it first expands to 112×112×96, and after a depth-wise convolution layer, it decreases to 56×56×96. After the second point-wise convolution layer, it decreases to 56×56×24. As the feature vector passes through the bottleneck, its dimensions increase from 16 to 96 and then reduce to 24. This structure ensures that the depth-wise convolution within the bottleneck captures rich feature information while effectively reducing the memory required for model training. To address the feature loss issue when compressing high-dimensional features to low-dimensional features, MobileNet V2 replaces the ReLU6 non-linear activation function in the second pointwise convolution layer with the linear operation Linear while keeping ReLU6 unchanged in other positions, thus ensuring feature information diversity.

Similar to MobileNet V2, ShuffleNet V2 adopts depth-wise separable convolution but also adds channel shuffling and grouped convolution operations. When training the image modality with ShuffleNet V2, the input image first passes through a convolutional layer, a max-pooling layer, three stage modules, and then another convolutional layer to obtain the image feature vector. In Stage 2, Stage 3, and Stage 4, a downsampling operation with Stride = 2 is first performed, followed by different numbers of depth-wise separable convolutions and grouped convolutions. For training, the input vector is divided into two branches, branch1 and branch2. The first inverted residual module performs downsampling on both branches. In subsequent inverted residual modules, only branch2 undergoes depth-wise separable convolution operations. After completing the inverted residual module, the vectors of the two branches are concatenated and channel shuffling is performed to ensure the mutual interaction of information between the two branches.

#### 2.6.2 Text branching in the multimodal framework

The text branch selected TextCNN, which performed well in the single-text modality comparative experiments. It is a convolutional neural network designed for text classification, and its structure is depicted in **Figure 1E**. The text modality data are derived from the structured citrus disease features glossary, and data enhancement methods are embedded into the textual descriptions at the time of image enhancement to ensure correspondence of the image-text pairs. When training the text with TextCNN, word indices of the input text sequence are first mapped to fixed-dimensional word vectors through an embedding layer. Then, the word vectors pass through a convolutional layer. The width of the convolutional kernel is the same as the dimension of the word vector, and its height corresponds to each value in kernel_sizes. This convolution operation can capture local word sequence features. Then, the ReLU activation function is applied to enhance the network’s non-linear capability. Next, a max-pooling operation is applied to each convolution output, and the maximum value from each feature map is selected as the output. This process retains the most significant features extracted by each convolution kernel. Finally, the features extracted by each convolution kernel are concatenated to obtain the text feature vector.

#### 2.6.3 Feature fusion

The features extracted from the different modalities are fused at the feature layer. For the ith object, the features extracted from the image and text are denoted as $f_{img}\left(x_{img}^{i}\right)$ and $f_{text}\left(x_{text}^{i}\right)$ , respectively. The fused feature is represented by $f_{\emptyset }\left(x_{fusion}^{i}\right)$ . First, the image and text features were projected into low-dimensional space using a projection matrix. The two projected vectors were then concatenated and passed through a convolutional layer for feature learning to obtain the fused feature output. C represents the feature vector learned through the convolutional layer. The convolution uses a 1×1 kernel with a stride of 1. The 1×1 convolution itself does not change the size of the feature map but can reduce the dimension of the vector. During the dimension reduction process, interaction information can be learned between multiple channels. The feature mapping function M is represented in equation (1), and the fused feature is represented in equation (2).


(1) $$M\left(x\right)=W_{x}\cdot x$$

(2) $$f_{\emptyset }\left(x_{fusion}^{i}\right)=C\left(F\left(M\left(f_{img}\left(x_{img}^{i}\right)\right),M\left(\;f_{text}\left(x_{text}^{i}\right)\right)\right)\right)$$

where *F*denotes the feature vector concatenation function and *C*represents the feature vector after convolutional layer processing. $W_{x}\in \left\{N\times D_{x}\right\}\;$ is the projection matrix for the features, *D_x_* indicates the dimension of the input feature *x*, and *N* signifies the dimension after projection.

The loss function employs the Cross-entropy loss function. In this context, *x*_2_ is the predicted result and represented as a vector $x=\left[x_{1},x_{2},\ldots ,x_{n}\right]$ . The number of elements in this vector is equivalent to the number of categories. The variable “class” indicates the true label of the sample. For instance, if the sample belongs to the second category, then *class* = 2. Consequently, $x_{\left[class\right]}$ refers to *x*_2_. This implies that the second element was extracted from the predicted result vector, which corresponds to the predicted value of the true category, as depicted in equation (3).


(3) $$loss\left(x,class\right)=-log\left(\frac{e^{x_{\left[class\right]}}}{\sum_{j}e^{x_{j}}}\right)=-x_{\left[class\right]}+log\left(\sum_{j}e^{x_{j}}\right)$$

### 2.7 Experiment environments

The research and control experiments were conducted in an Ubuntu 20.04 environment (processor: Intel core i9 9820X; RAM: 64G; graphics card: NVIDIA RTX A4000 16G DDR6). The deep learning framework Pytorch was used along with Cuda10.1 for training. In the experimental design and comparison processes, the batch size for the training and validation sets was set to 32. Based on the characteristics and convergence of the modalities data, the number of iterations for the single image modality was set to 200 while the preset number of iterations for the single text modality was set to 50. The multimodal model converged rapidly, and early stopping was applied to prevent overfitting.

### 2.8 Evaluation indices

This research compared the models from four perspectives: accuracy, precision, recall, and F1. The specific calculation methods are presented in equations (4–7).


(4) $$Accuracy=\frac{TP+TN}{TP+TN+FP+FN}$$

(5) $$Precision=\frac{TP}{TP+FP}$$

(6) $$Recall=\frac{TP}{TP+FN}$$

(7) $$F1=\frac{TP}{TP+FN}$$

where TP is the number of true positive samples, FP is the number of false positive samples, and FN is the number of false negative samples.

## 3 Results

### 3.1 Comparison of image modality models

**Figure 4:**
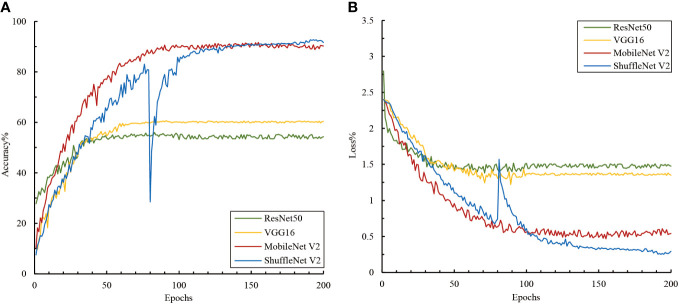
Accuracy and loss variations among the image models. **(A)** Accuracy and **(B)** loss.

A comparison of the performance of the deep and lightweight networks in the single-image modality classification tasks showed that the lightweight neural networks exhibited better feature extraction and classification performance on small-sample natural background image datasets (**Figure 4**). Although deep neural networks such as VGG16 and ResNet50 converged quickly during training, they only achieved classification accuracies of 54.33% and 60.39%, respectively. In contrast, MobileNet V2 and ShuffleNet V2, the two lightweight networks, achieved high classification accuracies of 90.19% and 91.63% respectively, despite converging at a slower rate. This might be because VGG16 and ResNet50 have complex structures that allow them to rapidly capture features in the data. However, this depth might also render them more susceptible to limitations imposed by data volume, impacting accuracy. The results suggest that lightweight networks are more suitable for small sample data. Their simplified structures and various strategies, such as depth-wise separable convolutions and channel shuffling, enhance their adaptability to complex background data.

Additionally, we observed noticeable fluctuations in ShuffleNet V2 during training. A local minimum or plateau area was encountered around the 80^th^ epoch, and it was subsequently adjusted using the algorithm to escape this state and achieve better convergence. Although ShuffleNet V2 exhibited fluctuations around the 80^th^ epoch, it could converge normally and reach a high accuracy afterward. This demonstrated the robustness and self-recovery capabilities of the proposed model.

We further analyzed the ability to recognize different disease categories based on a single-image modality at a finer granularity. The average precision, recall, and F1 values for all image networks presented ranges of 58.12–84.89%, 57.21–85.72%, and 56.82–85.62%, respectively (**Table 2**). The lightweight network ShuffleNet V2, which boasts high generalization capability and computational efficiency, performed the best, and its confusion matrix of validation and training results is presented in **Figure 4**. An analysis of the convolutional neural network feature extraction and classification results for the 11 citrus leaf sample images revealed significant differences between different category samples. The four models perform well in extracting features for citrus scab (CSC), citrus greening (CGR), citrus canker (CCA), healthy leaves (CK), and citrus sooty mold (CSM). However, their classification results for citrus greasy spot (CGS), citrus magnesium deficiency (CMD), and citrus anthracnose (CAN) were less satisfactory, with F1 average values of <70%. Further examination of the image dataset revealed that several disease categories with higher error rates contained background noise, including non-disease features, such as fingers and fruits, which may confuse the model’s classification of these samples. Additionally, the disease categories CGS and CAN included some early stage symptoms, which increased the difficulty of distinguishing between the more challenging diseases and subsequently affected the classification performance.

**Table 2:** Eleven classification results of the image modal networks.

Disease category	VGG16			ResNet50			MobileNet V2			ShuffleNet V2		
	Precision(%)	Recall(%)	F1(%)	Precision(%)	Recall(%)	F1(%)	Precision(%)	Recall(%)	F1(%)	Precision(%)	Recall(%)	F1(%)
CK	72.55	67.27	69.81	57.89	60.00	58.93	92.00	83.64	87.62	88.68	85.45	87.04
CGR	59.26	65.31	62.14	68.18	61.22	64.52	90.20	93.88	92.00	97.87	93.88	95.83
CCA	56.94	73.21	64.06	73.91	60.71	66.67	92.00	82.14	86.79	85.96	87.50	86.73
CAN	60.78	56.36	58.49	57.89	40.00	47.31	74.24	89.09	80.99	79.41	98.18	87.80
CSC	77.78	77.78	77.78	75.00	73.33	74.16	87.80	80.00	83.72	97.56	88.89	93.02
CGS	40.63	26.00	31.71	46.15	36.00	40.45	75.47	80.00	77.67	96.55	56.00	70.89
CME	60.53	80.70	69.17	43.88	75.44	55.48	75.41	80.70	77.97	84.75	87.72	86.21
CSM	68.42	56.52	61.90	60.00	65.22	62.50	81.48	95.65	88.00	82.69	93.48	87.76
CND	57.58	76.00	65.52	51.72	60.00	55.56	97.67	84.00	90.32	80.00	80.00	80.00
CMD	58.70	45.76	51.43	48.78	33.90	40.00	82.35	71.19	76.36	83.58	94.92	88.89
CID	72.09	59.62	65.26	55.93	63.46	59.46	85.19	88.46	86.79	78.43	76.92	77.67
Avg.	62.30	62.23	61.57	58.12	57.21	56.82	84.89	84.43	84.39	86.86	85.72	85.62

### 3.2 Comparison of the text modality models

**Figure 5:**
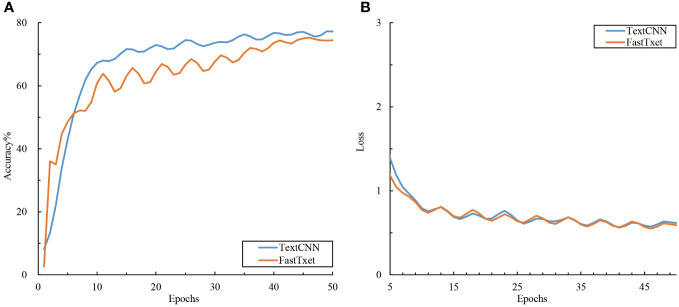
Accuracy and loss variations among the text models. **(A)** Accuracy and **(B)** loss.

The accuracy and loss curves during the training of the text branch training set are shown in **Figure 5**. An analysis of the performance of the different text models in the citrus disease classification task showed that the two models in the text branch displayed approximately the same performance (**Figure 5**) and converged quickly around the 10^th^ epoch. The final training accuracies of TextCNN and fastText were 77.22% and 74.42% respectively, with both showing reduced loss values of approximately 0.6. A comparison with the image modality model showed that the text modality model converged faster, with its accuracy lying between that of the lightweight and deep convolutional image networks. This might be attributed to the fact that text data are more structured than are image data; thus, the features can be more quickly learned by the model. However, the relatively lower accuracy might indicate that the information contained in the text data is not as rich as that in the image data for the task of citrus disease classification.

The precision, recall, and F1 values of the two text networks were between 78.36% and 82.83% (**Table 3**). TextCNN, which benefits from the features of both convolutional and recurrent neural networks, exhibited superior performance relative to fastText. When extracting text features and classifying the 11 categories of citrus leaf samples, the performance between the different sample categories varied significantly. Specifically, for categories such as CK, CSM, CMD, and Citrus iron deficiency (CID), the text models effectively extracted features with F1 scores >95%. However, for the CGS and CCA categories, the classification results of the text models were suboptimal, with F1 scores<50%. The analysis of the disease text dataset showed that certain descriptors of disease spots, such as “yellow,” “smooth,” and “randomly distributed,” frequently occurred in the descriptions of several disease categories. Conversely, highly distinctive descriptive phrases such as “asymptomatic” for healthy leaves and “volcano-like spots” for ulcers were missing from these two disease categories. Such descriptors have a stronger discriminatory power for text classification. In summary, although text modality performs relatively well in classifying citrus diseases, it faces challenges such as variations in sample categories and disparities in descriptive phrases.

**Table 3:** Eleven classification results of the text modal networks.

Disease category	FastText			TextCNN		
	Precision (%)	Recall (%)	F1 (%)	Precision (%)	Recall (%)	F1 (%)
CK	100.00	100.00	100.00	100.00	100.00	100.00
CGR	78.79	98.11	87.39	78.79	98.11	87.39
CCA	43.14	44.90	44.00	43.14	44.90	44.00
CAN	84.78	88.64	86.67	84.44	86.36	85.39
CSC	70.69	78.85	74.55	70.69	78.85	74.55
CGS	61.11	18.97	28.95	57.89	18.97	28.57
CME	48.65	81.82	61.02	48.65	81.82	61.02
CSM	100.00	100.00	100.00	100.00	100.00	100.00
CND	96.97	71.11	82.05	96.97	71.11	82.05
CMD	100.00	98.08	99.03	100.00	98.08	99.03
CID	100.00	100.00	100.00	100.00	100.00	100.00
Avg.	80.38	80.04	78.51	80.05	79.84	78.36

**Figure 6:**
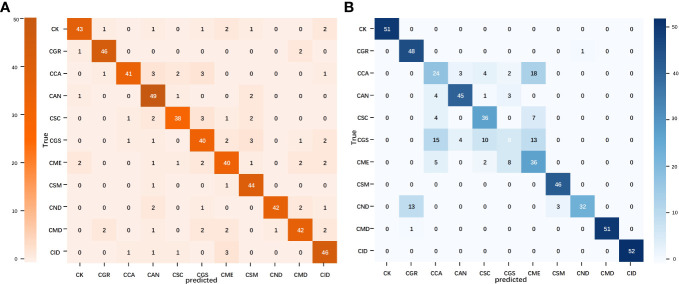
Confusion matrix of the image and text modality optimal models. **(A)** MobileNet V2 confusion matrix and **(B)** TextCNN confusion matrix.

A confusion matrix is a tool for visualizing and quantifying the relationship between predicted results and actual labels. The confusion matrices of the best models for the image and text modalities are shown in **Figure 6**. In the confusion matrix for MobileNet V2, misclassifications were scattered in almost every category, suggesting that the image model has greater complexity and learning capacity and may have more flexibility when processing samples compared with the other models, thus enabling it to learn features more evenly across different categories. Compared with the image modality, misclassifications in the TextCNN confusion matrix were relatively concentrated but showed a higher error rate for individual points. This may be related to the limitations in text information when describing citrus leaf diseases as well as the lack of semantic richness, which increases the difficulty of accurately expressing and distinguishing all the features of different diseases. Consequently, the ability of TextCNN to extract features from certain citrus disease categories was comparatively weaker than that of MobileNet V2. In summary, the image and text modalities exhibited different strengths and limitations in classifying citrus leaves. A comparative analysis of single-modality models provided crucial insights and directions for subsequent modal fusion.

### 3.3 Effect of different datasets on classification performance

**Figure 7:**
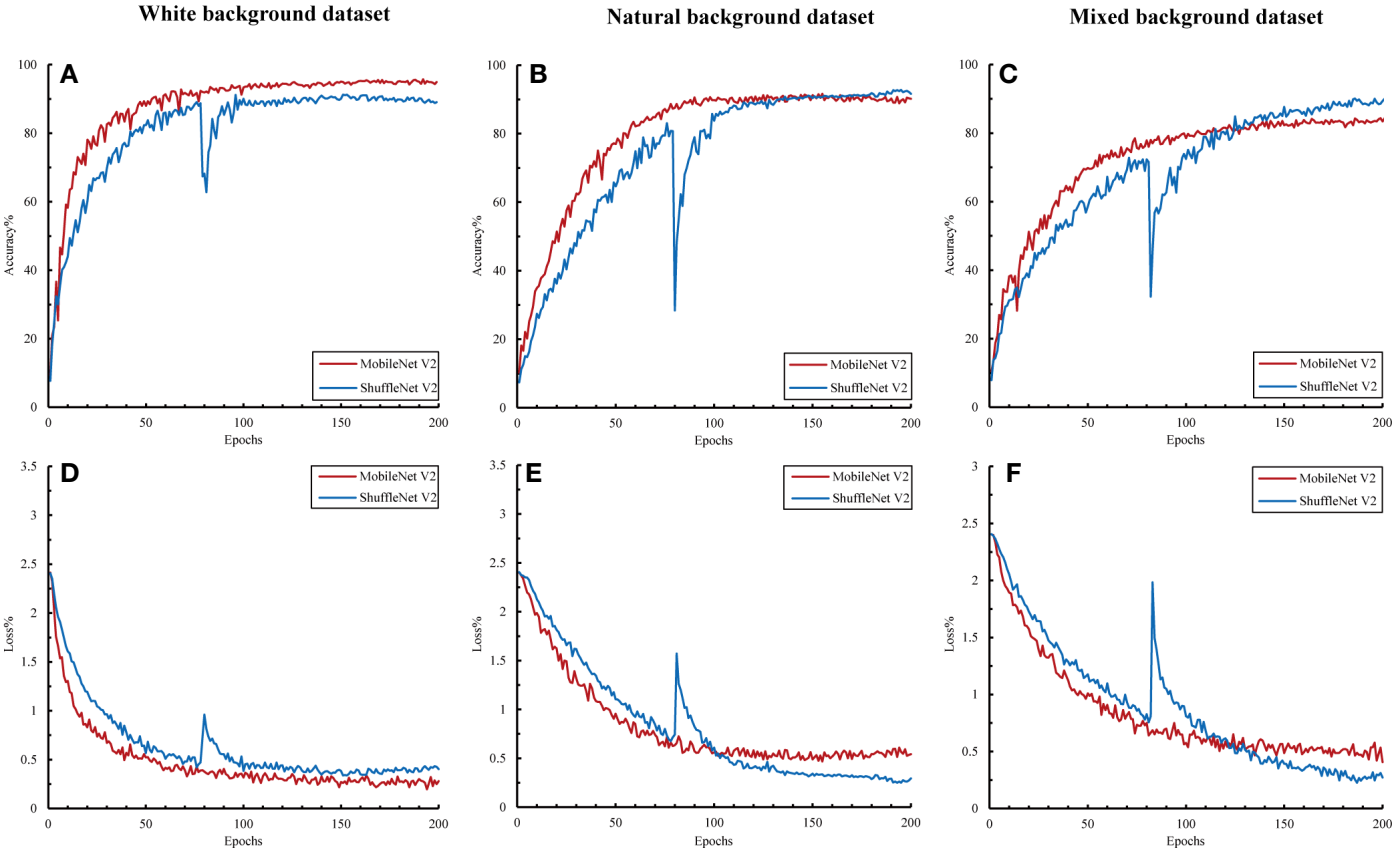
Accuracy and loss variations among the different datasets. **(A, D)** Accuracy and loss in the white background dataset; **(B, E)** accuracy and loss in the natural background dataset; and **(C, F)** accuracy and loss in the mixed background dataset.

The accuracy and loss curves for model training using different datasets are shown in **Figure 7**. An analysis of the performance of different datasets in the citrus disease classification task revealed that the citrus disease samples from the white background dataset achieved the highest classification accuracy of 89.61% and 94.76%, respectively, with the loss function stabilizing at<0.5. As the complexity of the sample background increased, the classification accuracy showed a decreasing trend, with the accuracy for the natural and mixed background data falling between 83.45% and 91.63%. This suggests that a simplified background can assist the model in more easily identifying the target, thereby improving accuracy. However, in natural and mixed-background datasets, a complex background might introduce considerable irrelevant information and noise, such as shadows and light variations. These factors could interfere with the model’s ability to extract key features. Nevertheless, the model still demonstrated a relatively high accuracy under these complex backgrounds, indicating that the selected models are robust and adaptable.

In the unified natural background test set, the models trained on different datasets showed significant differences (**Table 4**). The test accuracy of the model trained on the white background dataset was<21%, which is significantly lower than its training accuracy. This indicates that the model overfilled on a simplified and non-disturbed background and failed to fully learn the clutter features, resulting in a poor generalization ability. Conversely, the models trained on the natural and mixed-background datasets presented similar accuracy as their training accuracy. The test accuracy of ShuffleNet V2 reached 92.31%, surpassing its performance in the training and validation sets. This suggests that sample diversity helps the model learn to distinguish target objects from key features during training and resist background interference, thereby significantly improving the robustness and generalization of the model.

**Table 4:** Test set results in the image modal networks.

Dataset class	MobileNet V2			ShuffleNet V2		
	Precision (%)	Recall (%)	F1 (%)	Precision (%)	Recall (%)	F1 (%)
White	21.00	17.17	17.36	19.20	15.12	16.03
Natural	84.89	84.43	84.39	86.86	85.72	85.62
Mixed	85.11	84.18	84.37	92.31	91.72	91.81

**Figure 8:**
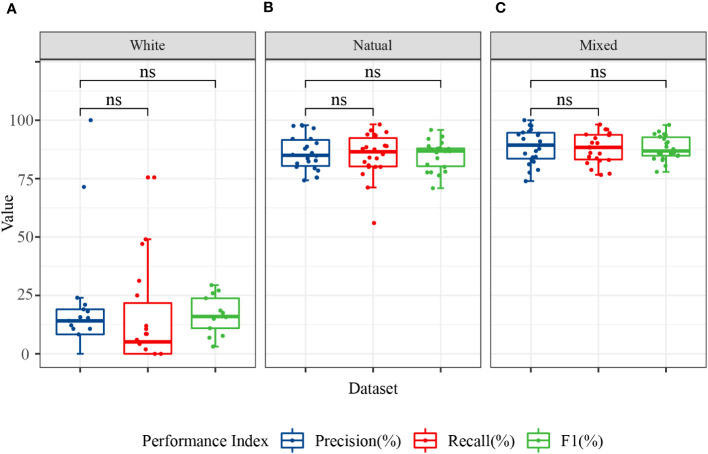
Classification performance of the image modality models for 11 categories. The data in the figure represent the precision, recall, and F1 of the 11 citrus samples classified using MobileNet V2 and ShuffleNet V2 models. **(A)** Precision, recall, and F1 in the white background dataset; **(B)** precision, recall, and F1 in the natural background dataset; and **(C)** precision, recall, and F1 in the mixed background dataset. ns, no significance.

The ability of the models to recognize different disease categories under different training datasets was analyzed at a finer granularity. Box plots for the precision, recall, and F1 values of each disease category showed that the models exhibited greater balance when trained with mixed background datasets (**Figure 8**). Although the test accuracy of the mixed dataset was slightly lower than that of the complex background, its performance in recognizing different disease categories was more balanced, with no particular category showing significantly lower recall or F1 values. This finding may be related to the ability of the mixed dataset to promote learning of target features related only to the disease and the capture of auxiliary information highly correlated with the background. Although a slight decrease in accuracy may have occurred, the adaptability to complex backgrounds and disturbances was stronger.

In conclusion, different dataset backgrounds, model structures, and training strategies can influence the model’s classification performance in real-world applications. The single-image modality analysis showed that the use of a mixed dataset combined with a lightweight neural network can yield a superior classification accuracy, generalization capability, and category balance.

### 3.4 Comparison of the multimodal models

**Figure 9:**
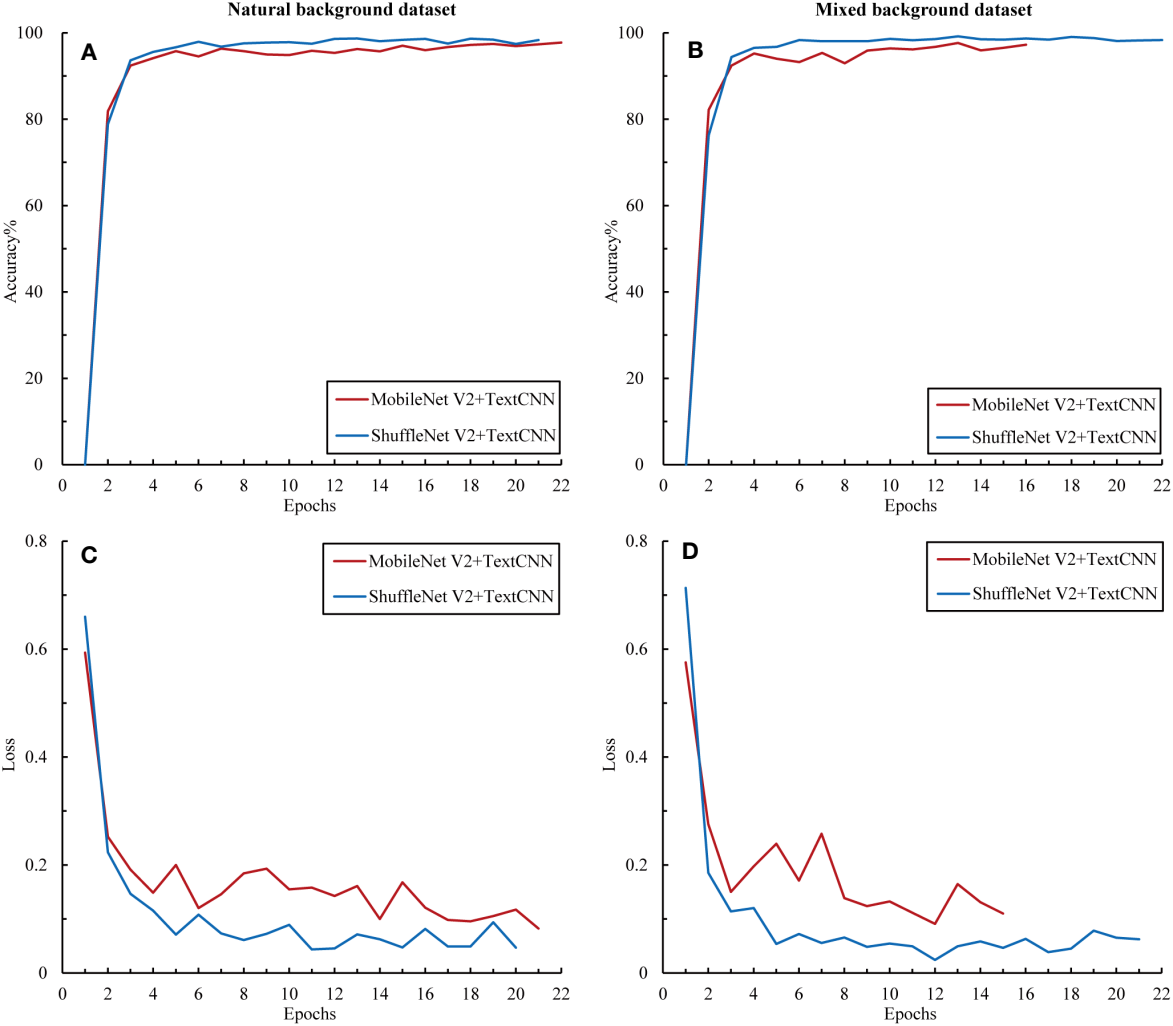
Accuracy and loss variations among the multimodal models. **(A)** Accuracy in the natural background dataset, **(B)** loss in the natural background dataset, **(C)** accuracy in the mixed background dataset, and **(D)** loss in the mixed background dataset.

To further explore the complementarity between the image and text modalities, we investigated the potential of feature fusion strategies to address the classification problem of citrus diseases with complex backgrounds. The accuracy and loss curves of the fusion model during the training process for the training set are shown in **Figure 9**. Compared with the previous unimodal results, the results of the multimodal network exhibited significant improvements. The fused networks MobileNet50 + TextCNN and ShuffleNet V2 + TextCNN both achieved training accuracies of over 95% on the two datasets and reduced the loss to below 0.2. Among them, ShuffleNet V2 + TextCNN yielded the best training results on the mixed-background dataset, with an accuracy of 98.34%. Moreover, the multimodal network converged within 10 epochs. To prevent overfitting, we applied early stopping. The results show that all models stopped within 22 epochs, indicating marked acceleration in training. No further fluctuations were observed, indicating that the multimodal network fully uses the complementarity between the image and text modalities. The cross-transfer of information and comprehensive use of features enhanced the network’s ability to extract and recognize different characteristics of citrus diseases.

The precision, recall, and F1 of the two fusion networks ranged from 97% to 99%, thus showing excellent performance (**Table 5**). Notably, the multimodal model achieved satisfactory results in classifying each type of citrus sample. In the two datasets, the F1 score for each sample type exceeded 90%. This performance enhancement may be attributable to the fact that after fusing unimodal information, the model can fully use the complementarity between this information and the cross-validation effect, helping the model to capture each category’s features more accurately and reducing the likelihood of misclassification.

**Table 5:** Eleven classification results of Multimodal networks.

Disease category	Natural Background Dataset						Mixed Background Dataset					
	MobileNet V2+TextCNN			ShuffleNet V2+TextCNN			MobileNet V2+TextCNN			ShuffleNet V2+TextCNN		
	Precision (%)	Recall (%)	F1 (%)	Precision (%)	Recall (%)	F1 (%)	Precision (%)	Recall (%)	F1 (%)	Precision (%)	Recall (%)	F1 (%)
CK	1.00	1.00	1.00	1.00	1.00	1.00	1.00	1.00	1.00	1.00	1.00	1.00
CGR	0.96	0.96	0.96	1.00	0.96	0.98	0.98	0.92	0.95	1.00	0.96	0.98
CCA	1.00	1.00	1.00	0.98	0.96	0.97	0.90	0.92	0.91	0.94	0.96	0.95
CAN	0.98	0.98	0.98	1.00	1.00	1.00	0.98	0.85	0.91	0.98	0.94	0.96
CSC	1.00	0.96	0.98	1.00	1.00	1.00	1.00	0.98	0.99	0.98	0.98	0.98
CGS	0.94	0.98	0.96	0.96	0.98	0.97	0.85	0.96	0.90	0.88	0.96	0.92
CME	0.96	0.96	0.96	1.00	1.00	1.00	1.00	1.00	1.00	1.00	0.94	0.97
CSM	1.00	1.00	1.00	1.00	0.98	0.99	1.00	1.00	1.00	1.00	1.00	1.00
CND	1.00	0.94	0.97	0.96	1.00	0.98	0.93	0.98	0.95	0.96	1.00	0.98
CMD	0.94	1.00	0.97	0.98	1.00	0.99	1.00	1.00	1.00	1.00	1.00	1.00
CID	1.00	1.00	1.00	1.00	1.00	1.00	1.00	1.00	1.00	1.00	1.00	1.00
Avg.	0.98	0.98	0.98	0.99	0.99	0.99	0.97	0.96	0.96	0.98	0.98	0.98

**Figure 10:**
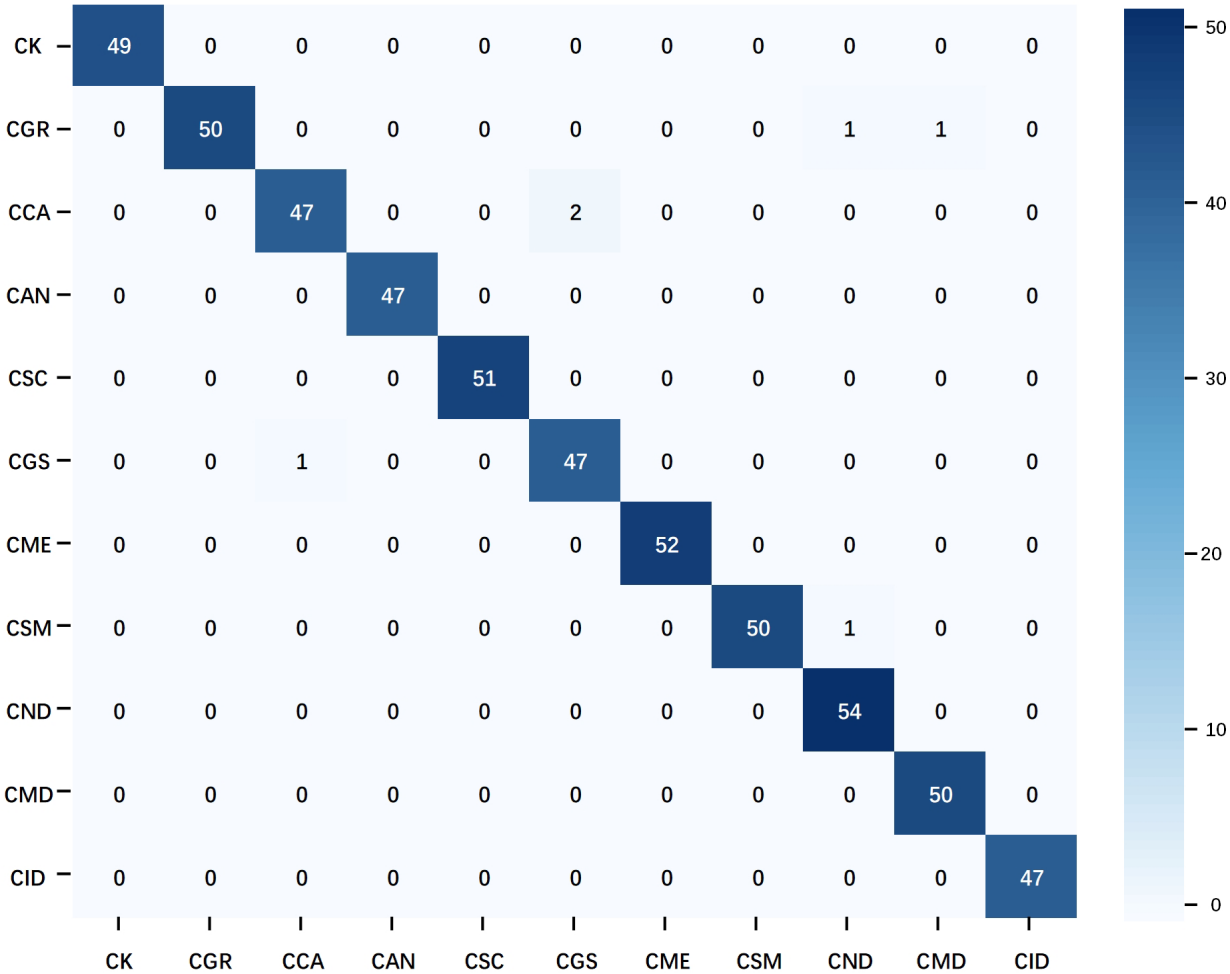
Confusion matrix of the multimodal optimal model. The data used in the matrix are the test results of ShuffleNet V2 on natural background images after training on the mixed dataset.

The confusion matrix of the optimal multimodal model–ShuffleNet V2 + TextCNN–further confirms the superiority of the multimodal strategy (**Figure 10**). Compared with the unimodal model, this model reduced the number of misclassifications and avoided the concentration of misclassifications in specific categories, demonstrating better balance and generalization. Only a few groups had errors, which did not exceed 2. Overall, when handling the citrus disease classification task in complex backgrounds, the multimodal strategy displayed higher robustness and stability than the unimodal strategy.

## 4 Discussion

Multimodal feature fusion has become a popular research direction in the field of plant disease classification. From the perspective of information complementarity, a single modality, such as image or text, has inherent limitations. While the image modality can capture rich visual features, it may be affected by factors such as lighting, shadows, and background noise (Huang et al., 2022). Moreover, although the text modality can provide semantic and contextual information about the disease (He and Peng, 2020), it might lack sufficient details to describe certain subtle visual features. These limitations can lead to classification inaccuracies. Combining multi-source data, such as image and text (Feng et al., 2022), image and hyperspectral data (Yang et al., 2021), or image and sensor information (Zhao et al., 2020), leverages the strengths of each modality and is beneficial in improving classification accuracy and model robustness. In our experiments, we observed a clear advantage of multimodal joint analysis. The accuracy of ShuffleNet V2+TextCNN using the mixed dataset reached 98.33%, representing an improvement of 9.78% and 21.11%, compared with those of single image and single text modalities, respectively. This is consistent with previous results showing that the complementary information from multimodal data could enhance object detection capabilities, thus demonstrating the significant advantage of multimodal strategies in handling complex backgrounds and noisy data (Liu et al., 2020). Second, from the perspective of data fusion, feature fusion helps to enhance multimodal interaction and generalization performance, compared with decision fusion. Combining multi-source data at an early stage allows the model to better understand and distinguish complex backgrounds and noise, thereby improving classification accuracy (Yang et al., 2021). In contrast, decision fusion is typically performed at the later stages of the model and might not fully capture the fine-grained feature interactions between modalities. Moreover, classifying diseases using decision fusion might require a high classification confidence level from one modality (Wang et al., 2021a). Therefore, multimodal feature fusion has a more pronounced advantage in tasks with complex and varied backgrounds, especially in scenarios requiring cross-modal collaboration, and showed promising results in our experiments.

Sample diversity has always been regarded as a key factor in the field of deep learning. However, due to the challenges of dataset preparation, its importance is often underestimated. Previous studies on citrus disease identification and classification preferred to use datasets with a single background (Barman et al., 2020; Yang et al., 2021; Dananjayan et al., 2022), with few utilizing natural background datasets (Xing et al., 2019). In addition, the disease categories and scenarios were relatively limited. The text involves 4 scenarios and 11 types of samples, rendering this citrus disease classification task with small samples, multiple scenarios, and multiple categories a higher challenge for model recognition performance. To address this, we designed a dataset with diverse backgrounds, including white, natural, and mixed backgrounds. This design has shown significant advantages in enhancing the model’s generalization capabilities and preventing data leakage issues. A diversified training set provides the model with a broader data distribution, enabling it to better handle unknown data. Studies have shown that training with diverse data helps the model to capture the underlying structures and patterns of the data, resulting in better performance in real-world applications (Hernandez and Lopez, 2020). In this study, training with natural and mixed backgrounds significantly improved the model’s test accuracy and led to better classification balance, thereby demonstrating the importance of sample diversity in enhancing the model’s generalization capabilities in real complex backgrounds. Moreover, data in the same dataset often come from the same location, the same environment, or even the same plant. This sample autocorrelation might pose a risk of data leakage (Stock et al., 2023). In this study, cross-validation of the dataset effectively reduced the potential for data leakage and overfitting, ensuring the fairness and authenticity of model evaluation and effectively ensuring the model’s generalization capabilities on natural background data.

The effective application of knowledge has demonstrated undeniable value in enhancing model accuracy and credibility. To improve the comprehensiveness, objectivity, and efficiency of text description, expert knowledge was fully utilized and a glossary of citrus disease characteristics was innovatively constructed to provide a new approach to text description. The construction of the feature word list allows describers to choose from 12 categories of feature words, comprehensively covering information such as leaf color, leaf morphology, affected parts, covering features, yellowing characteristics, and lesion features, effectively avoiding omissions or errors. Notably, in subsequent practical applications, users without a professional knowledge background can easily select highly specialized and specific phrases such as “netted chlorosis,” “leaf vein corking,” and “volcano-like lesions,” further enhancing the professionalism and reliability of the text modality. Zhou’s research also indicates that the application of domain knowledge is beneficial for improving classification accuracy and the interpretability of the model’s inference process (Zhou et al., 2021).

This study achieved satisfactory results in the citrus disease classification task with complex backgrounds by leveraging the complementary information of image-text multimodal and the advantages of sample diversity. However, there are still some shortcomings. First, although this experiment explored the feature fusion of image-text multimodal, the fusion strategy is relatively simple and may not fully mine the potential association information between image and text modalities. Further research on more advanced fusion strategies, such as attention mechanisms and multi-task learning, can better utilize the complementary information between images and text. Second, the interpretability and explainability of the model in this experiment require improvement. To enhance the credibility and application value of the model, further research on model interpretability methods is needed. This will help researchers and agricultural practitioners to better understand how the model works, which is of great significance for improving productivity and reducing economic losses in the citrus industry.

## 5 Conclusion

The image-text multimodal deep learning method proposed in this study combined text and image features with domain knowledge to fully characterize the features of various diseases and accurately identify and infer the main types of citrus diseases. Even when dealing with small-sample and multi-background noise datasets, this method achieved a high classification accuracy and generalization performance. Moreover, by constructing a structured feature word table as prior knowledge for text information preparation, this study significantly reduced the volume and preprocessing difficulty of the text modality. The inclusion of domain knowledge also provided prediction results with higher credibility. Taken together, the multimodal deep learning method proposed in this study can effectively extract and integrate features from multiple data sources and domain knowledge, thereby achieving precise identification of citrus diseases in complex backgrounds. This provides a more reliable basis for making decisions regarding the precise application of biological and biochemical control strategies in production.

## Data availability statement

The original contributions presented in the study are included in the article/**Supplementary Material**. Further inquiries can be directed to the corresponding author.

## Author contributions

XQ: Conceptualization, Investigation, Writing – original draft. HC: Methodology, Software, Writing – original draft. PH: Formal Analysis, Writing – review & editing. DZ: Data curation, Software, Writing – original draft. TG: Writing – original draft, Data curation, Software. CP: Investigation, Writing – original draft. ZL: Writing – review & editing, Formal Analysis. YL: Writing – review & editing, Investigation. JC: Methodology, Writing – review & editing. SW: Conceptualization, Writing – review & editing.
