# Supplementary material for: Detection of citrus diseases in complex backgrounds based on image–text multimodal fusion and knowledge assistance
Source: Front Plant Sci. 2023 Nov 27;14:1280365. doi: 10.3389/fpls.2023.1280365 (PMC10711083; doi:10.3389/fpls.2023.1280365)
Supplement: Supplementary file 1 [file Table_1.docx]

Supplementary Material

# Supplementary Table

Supplementary Table 1. Structured citrus disease features glossary

| **No.** | **Category** | **Feature Description** |
| --- | --- | --- |
| **1** | **Leaf Color 叶色** | Deep Green 深绿 |
|  |  | Light Green 浅绿 |
|  |  | Yellow 黄色 |
|  |  | White 白色 |
| **2** | **Leaf Morphology 叶片形态** | **Normal** 正常 |
|  |  | **Curled** 卷曲 |
| **3** | **Affected Areas 为害部位** | **Leaf front side** 叶面 |
|  |  | **Leaf back side** 叶背 |
|  |  | **Asymptomatic** 无症状 |
| **4** | **Covering Features 覆盖物特征** | **White Powdery Spots or Layer** 白色粉点或粉层 |
|  |  | **Black Mold Spots or Layer** 黑色霉点或霉层 |
| **5** | **Chlorosis Region 黄化区域** | **Uniform Yellowing** 均匀黄化 |
|  |  | **Randomly Dispersed** 随机分散 |
|  |  | **Veined and randomly dispersed** 叶脉和随机分散 |
|  |  | **Near Petiole 靠近叶柄** |
|  |  | **Near Leaf Margin** 靠近叶缘 |
|  |  | **Leaf pulp Only** 仅叶肉 |
|  |  | **Veins Only** 仅叶脉 |
| **6** | **Chlorosis Features 黄化特征** | **Interveinal Netted Chlorosis 脉间**网状失绿 |
|  |  | **Inverted V-shaped Chlorosis** 倒V字型失绿 |
|  |  | **Vein Corking** 叶脉木栓化 |
| **7** | **Lesion Shape 病斑形态** | **Subcircular** 近圆形 |
|  |  | **Irregular** 不规则 |
| **8** | **Lesion Count 病斑数量** | **Less than 3**小于3个 |
|  |  | **4–20** 4**–**20个 |
|  |  | **More than 20**大于20个 |
| **9** | **Lesion Size 病斑大小** | **Less than 1 mm** 小于1 mm |
|  |  | **1–3 mm** 1–3 mm |
|  |  | **3–5 mm** 3–5 mm |
|  |  | **5–15 mm** 5–15 mm |
|  |  | **Larger than 15 mm 大于**15 mm |
| **10** | **Lesion Distribution 病斑分布** | **Randomly Dispersed** 随机分散 |
|  |  | **Near Leaf Margin** 靠近叶缘 |
|  |  | **Near Veins** 靠近叶脉 |
|  |  | **Near Petiole** 靠近叶柄 |
| **11** | **Lesion Color 病斑颜色** | **Yellow** 黄色 |
|  |  | **Black** 黑色 |
|  |  | **Gray-White** 灰白色 |
|  |  | **Light Brown** 浅褐色 |
|  |  | **Dark Brown** 深褐色 |
|  |  | **Red-Brown** 红褐色 |
|  |  | **Brown at the center and yellow at the edges** 中间褐色边缘黄色 |
|  |  | **Gray-white at the center and brown at the edges**中央灰白边缘褐色 |
| **12** | **Lesion Features 病斑特征** | **Flat** 平整 |
|  |  | **Convex** 凸起 |
|  |  | **Withered** 干枯 |
|  |  | **Ring-shaped** 轮纹状 |
|  |  | **Volcano-like** 火山口状 |
|  |  | **Greasy** 油脂状 |
|  |  | **Corked** 木栓化 |
|  |  | **Conical or Tumorous Protrusion** 圆锥状或瘤状凸起 |

Note: Features from classes 1–3 are general features. For covering, select features from class 4. For chlorosis, select features from classes 5–6. For lesions, select features from classes 7–12. Notably, features from class 12 can be selected multiple times.
